# InCHARGE: Co-creating, implementing and evaluating interventions to utilize nurses’ competence and achieve person-centred fundamental care—A research protocol describing an action research approach

**DOI:** 10.1371/journal.pone.0304700

**Published:** 2024-07-02

**Authors:** Anna Hauffman, Elin Björk, Katarina Edfeldt, Camilla Fröjd, Anna-Karin Gunnarsson, Lena Nyholm, Therese Avallin, Eva Jangland

**Affiliations:** 1 Department of Surgical Sciences, Section of Nursing research, Uppsala University, Uppsala, Sweden; 2 Department of Surgery, Uppsala university hospital, Uppsala, Sweden; University of Verona, ITALY

## Abstract

**Aim and objectives:**

This research protocol presents an action research project with the aim to demonstrate the value of person-centred fundamental care to nurses and nurse managers in surgical care units to encourage a far-reaching change in this direction. The objectives are to describe this process and to evaluate the effects on missed nursing care and person-centred fundamental care.

**Methods:**

In a novel collaboration between nursing science and medical humanities the action research design will be used to interact with nursing staff and leaders in three surgical care units and design interventions with the purpose to affect the direction of nursing. Initially, the care units will be presented with interactive workshops including evidence-based education on person-centered fundamental care, person-centredness, nurse role responsibility and leadership. This will be followed by cocreation of interventions to stimulate person-centered fundamental care. The Fundamentals of Care framework will be used as the overarching theoretical framework. Data on missed nursing care, person-centred climate and person-centered fundamental care will be collected repeatedly from patient- and nursing stakeholders through interviews and validated questionnaires. Additionally, data from written reflections following clinical observations and focus group interviews will be included. The duration of the study will be approximately five years from ethical approval.

**Discussion:**

It has been previously reported that the current working environments of registered nurses are forcing them to ration their caring responsibilities, leading to a lack of fulfillment of patients’ fundamental care needs, with possible severe consequences for patients. The action research design helps researchers gain an understanding of the contextual factors important for forthcoming interventions, enabling reflective processes and cocreation of interventions with stakeholders. This may lead to feasible interventions and strengthen nursing leadership in the involved units.

## Introduction

It has been reported that the current working environments of registered nurses (RNs) are forcing them to ration their caring responsibilities. This leads to a shift within nursing where the widely ruling norm among RNs is to provide a task-oriented, fragmented nursing care leaving many of the patients caring needs aside [1]. This suggests a need for a new working culture that promotes learning, safety, quality, and person-centeredness and this protocol paper will describe a project with the purpose of encouraging care units in this direction.

### Background

A task-oriented, fragmented nursing care may negatively affect patients in several ways. Missed nursing care (MNC) is defined as any aspect of required patient care that is either delayed, incomplete, or absent [2, 3]. Worldwide, the occurrence of MNC is high, affecting fundamental care such as comforting and educating patients and family, pain management, skincare, changing patients’ position, oral hygiene, administering medication on time, et cetera [4, 5]. This has severe consequences for patient safety and the quality of care, including an increased risk of mortality [5–7]. The experiences of unmet needs among patients due to MNC have also been investigated, with results showing that patients are fully aware that their nursing care needs are not met, causing feelings of unsafety and loss of dignity while in hospital [4]. To ensure high-quality fundamental care and avoid MNC, nursing leadership at several levels in the healthcare system, from bedside to overall management, is vital. However, it is a well-known problem that healthcare organizations do not fully use RNs’ capacity and competence in contributing to the quality of care and patient safety (e.g., prevention of adverse events) [8, 9]. Furthermore, previous research reports that RNs are prone to quit their jobs when their competence is not fully utilized, when there is a lack of professional opportunities, or when their autonomy as RNs is restricted [10–12]. Meanwhile, the World Health Organization (WHO) reports a global shortage of 5.9 million nurses, predicting the number to be 9 million in 2030 [13] and following this an urgent need for improved capacity to educate, employ and retain nurses. The same report states that nursing leadership and governance are critical aspects of strengthening the nursing workforce. Accordingly, since approximately 90% of the nurses worldwide are women, future policy considerations are recommended to include enabling work environments (i.e., gender transformative leadership) [13].

In response to the concerns regarding the lack of fulfillment of patients’ fundamental care needs, the International Learning Collaborative (ILC) was formed in 2008, with the main purpose to generate evidence to improve the delivery of person-centred fundamental care worldwide. The work within the ILC has resulted in the Fundamentals of Care (FoC) framework [14] addressing the patient’s fundamental care needs. The conceptual framework comprises three interrelated dimensions: relationship, integration of care and context. The nurse-patient relationship is the core of the framework, and establishing a positive relationship with the patient requires; developing trust, focusing and giving the patient undivided attention, anticipating needs, knowing enough about the patient to act appropriately and evaluating the quality of the relationship. The second dimension focuses on integration of the patient’s physical needs (e.g., eating and drinking, rest and sleep, personal cleansing and dressing) and psychosocial needs (e.g., being involved and informed, dignity and privacy), and the importance of the nurse-patient relationship in recognizing and managing these needs (e.g., being empathetic, active listening). The final dimension of the framework refers to the context of care in which care is taking place. The context of care, including system- and policy-level factors (e.g., leadership, culture, governance and regulation), that hinder or enable the delivery of person-centred fundamental care [14, 15]. The framework has been translated into Swedish, including a cultural adaptation process, so it can be used to guide practice and change in the Swedish context [16]. A figure of the FoC framework (including additionally translations into a range of languages) can be found on the ILC’s website (https://ilccare.org/the-fundamentals-of-care-framework/). FoC is described as a point of care nursing theory [14] helping nurses and leaders navigate the practical aspects of nursing. Bedside nurses across several countries, address that the framework can be applied in clinical practice to reinforce leadership [17] and successful initiatives were leaders raised the awareness to the value of fundamental care in different collaborations are available [18–20]. Within the framework the term person-centered care is often used but not included in the definition [15].

Person-centredness has been shown to be helpful in avoiding MNC [21] improving the quality of care [22, 23] and increase nurse job satisfaction [24] but a person-centred culture does not ‘emerge naturally’ and is described as complex to implement since it deals with a need of a cultural shift to take place in clinical practice [25, 26]. The complexity of person-centred nursing is described in the mid-range theory of the Person-centred Nursing Framework [25]. The relationship between the constructs of the framework (i.e., contextual, attitudinal, and moral dimensions of humanistic caring practices) highlights the necessity of competent nurses who can manage the numerous contextual and attitudinal factors that exist within a care environment, and to engage in processes that keep the person at the center of caring interactions. The framework is underpinned by values of respect for persons (personhood), individual right to self-determination, mutual respect and understanding. Furthermore, it is underpinned by four core concepts that constitute person-centred nursing; being in relation, being in a social world, being in a place and being with self. These different perspectives provide a lens that can shape the way person-centredness is operationalized in practice. According to the framework person-centredness is enabled by cultures of empowerment that foster continuous approaches to practice development. Person-centredness does not simply entail focusing on an individual, but needs to consider the context from which the person is created [25]. Performing research on person-centred care (PCC) requires an approach to the research underpinned by the same values as described above, as well as awareness that such complex intervention will need creativity, strategic work and collaboration with stakeholders [25].

There is a need for research concerning the effects of person-centred fundamental nursing care, not only for the sake of the individuals in need of care, but also to prepare for future challenges within the nursing profession itself [27]. inCHARGE (“*Innovations to utilize nurses’ competence and achieve person-centered care–theory goes to practice”*) is a research program at Uppsala university with an interdisciplinary focus, integrating the scientific perspectives of nursing science and medical humanities with the overall aim to design innovations to improve patient care by optimizing nursing competence utilization. The inclusion of medical humanities contributes by offering a historical understanding of nursing development, both as a practice and as an identity. This paper will describe a project within inCHARGE using the action research method [28], based on the perspectives of stakeholders at the surgical department of a university hospital. Previous results show that the ever-changing patient volume at surgical care units and the adequacy of staffing are factors that influence the prevalence of MNC [29]. This makes it an especially important context for interventions.

#### Aims and objectives

The aim of this project is to demonstrate the value of person-centred fundamental care and achieve a far- reaching change in the direction of nursing in three surgical care units by using the action research approach.

The objectives are:

1. To investigate and report on the occurrence and cause of missed nursing care, person-centred climate and the fulfillment of fundamental care needs.
2. To understand and describe the contextual factors that enable and hinder the delivery of person-centred fundamental care.
3. To develop knowledge, commitment and ownership in staff and leaders for the delivery of person-centred fundamental care.
4. To design interventions to implement person-centred fundamental care.
5. To evaluate the AR process and the sustainability of interventions.

## Materials and methods

### Design

Action research design [26], including repeated measures, will be used, with three main phases: “Getting to know the context,” “Creating a context receptive to change,” and “Cocreating interventions.” The duration of the three main phases will be approximately five years from ethical approval of the first phase. The results from each phase will be used with reflexivity to shape the subsequent phase. The Fundamentals of Care framework will be used as the overarching theoretical framework in the project, highly appropriate in research concerning fundamental care needs and MNC [15–17]. During the process the research team will work with empowerment [30, 31] in relation to work role as the primary method in collaborations with stakeholders.

### Setting

The project will be undertaken at the surgical department of a university hospital comprising three care units, where patients are cared for following acute or planned admission due to vascular, endocrine, colorectal, esophageal/ventricle, or liver/bile/pancreatic illness, trauma, or transplantation. Each care unit has a ward manager and two assistant ward managers responsible for nursing care and staffing. Each patient is cared for by a RN and a nursing assistant (NA) at all times, while a surgeon visits the care unit during the day and on calls from the nurse. In addition, other health professions, such as physiotherapists and dieticians, work with patients on a consultant basis. Due to the department being short-staffed by about 20% of the number of RNs needed (in 2022), the patients are also cared for by agency nurses hired for shorter periods of time. Still, the care units have beds closed for admission due to a persistent shortage of nurses. The nurse shortage also means that patients have extended waiting time before admission, since surgery and other treatments are routinely postponed. Moreover, the potential emotional stress for waiting patients (including patients with cancer and other life-threatening diseases) and the progression of their illnesses increase the care needed when they are admitted to hospital, or may even lead to acute admission due to adverse events. Their care then requires even greater nursing competence to match their complex care needs.

### The research method

Action research (AR) was first described by Kurt Lewin in 1946 as a type of “social engineering” to achieve real change [28]. In his landmark paper, Lewin stated that “research that produces nothing but books will not suffice,” revealing the focus on social change in clinical practice. More recent AR methodologies [26, 32] are based on Lewin´s thoughts, encompassing the steps of planning, acting and observing, followed by reflection for a revised plan of the next action that needs to be taken in the context. These steps are performed cyclically without a set endpoint and encompass the involvement of stakeholders and their expertise thus achieving a shared understanding and ensuring that all contextual dimensions are included. To achieve changes, the focus in AR is on a long-term, systematic approach with a distinct focus on making the context receptive to change before attempts are made to implement interventions. In this preparation, the active participation of stakeholders and the creation of an ownership among them is vital. This cooperation has the potential to empower stakeholders, increase the credibility of the research as well as facilitate the implementation. In the forthcoming study stakeholders refer to the individuals directly involved in nursing actions, i.e., patients and staff (RNs, NAs) as well as the nursing management and the assistant head of department. In the group of staff an additional group of facilitators has been identified to collaborate with the research team regularly to reflect and help form and run the project. The researcher’s main task, aside from bringing the scientific methods, is to support stakeholders to achieve meaningful change and development in their context, while also producing scientific knowledge. In the forthcoming study the latter will involve studying the effects of the AR process by repeated measures of quantitative data as well as reflecting and reporting on the process itself by qualitative methods.

### Ethical considerations

Participation of both patients and staff will be voluntary and all participants will be asked to provide written informed consent. The data collection has been approved by the Swedish Ethical Review Authority (DNR 2022-01557-01) and will be carried out through questionnaires, structured interviews, and observations.

## Results

### Step 1

#### “Getting to know the context” (baseline)

**Fig 1:**
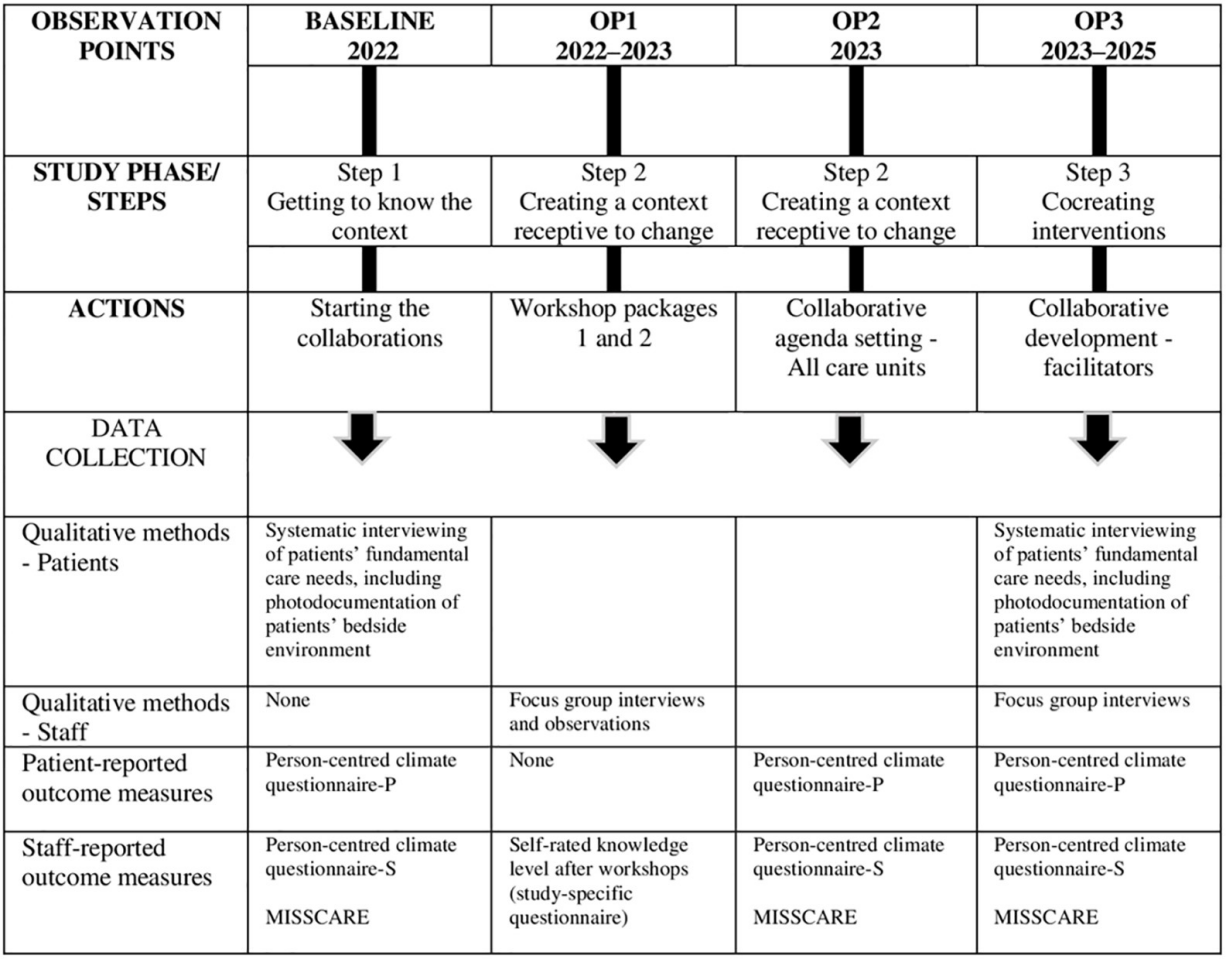
Planned observation points (OP) and actions within the inCHARGE project.

To gain a first understanding of the existing caring culture at the surgical care units and to guide the first collaborations, baseline data from patients and staff, i.e., stakeholders, will be collected by the research team (repeatedly in 2022–2025). The purpose of this data collection is to gain an understanding of patients own experiences regarding their fundamental care needs, the quality of the person-centred environment in the care units, and the prevalence of missed nursing care. A member of the research team will be present and responsible for data collection at each care unit. (For an overview of the planned observation points, see Fig 1).

### Participants

The stakeholders to be invited to participate include patients being cared for at the units, RNs NAs, ward managers, and assistant ward managers employed at the care units.

### Inclusion criteria

All RNs (n = 60) excluding agency nurses, NAs (n = 64), ward managers and assistant ward managers (n = 10) employed in the included departments will be invited to participate in conjunction with staff meetings. Since this investigation involves all employees above (n = 134), the sample size has not been calculated but assuming a 70% response rate the goal is to include questionnaire data from 100 nursing stakeholders. The admission rate is expected to allow for the equivalent number (n = 100) regarding data collection for patients.

All eligible patients aged 18 years or older being cared for at the units for at least 24 hours will be consecutively invited to participate by members of the research team. Data collection will be performed during 2 predetermined weeks. The exclusion criteria are cognitive impairment, infectious disease, Karnovsky performance status < 20, or inability to speak and understand Swedish.

### Data collection and analysis step 1

#### Fundamental care needs

To gain an understanding of patients’ own experiences regarding the fulfillment of their fundamental care needs while admitted to the care units, a structured interview guide built on the FoC framework has been constructed. The interview questions are short and cover patients’ fundamental physical and psychosocial care needs as well as the relational aspects of care. E.g. “Since admission, did you get the care and support you need concerning your personal hygiene needs?” Interviews will be conducted with the participating patients during their in-hospital stay (in an undisturbed environment). The answers will be marked (yes/no) in the interview guide and additional field notes will be taken. Interviews will be analyzed descriptively and by summative content analysis [33]. Patient stakeholders will be asked to participate consecutively, in conjunction with the invitation to answer questionnaires (below).

#### Person-centred climate

The quality of the person-centred climate in the care units will be measured with the validated Swedish version of the person-centred climate questionnaire, staff version [34] and patient version [35]. Questionnaires consist of 14 (staff) and 17 (patient) items with answers given on a six-point Likert-scale, from 1 = “Disagree completely” to 6 = “Agree completely.”

#### Missed nursing care

The Swedish version of the MISSCARE Survey [36] will be used to measure how often (part 1) and why (part 2) staff are not able to perform various nursing care measures. The first part consists of 24 nursing measures (e.g., oral care) with answers given on a five-point Likert-scale, from “always carried out” to “never carried out”. The second part consists of 17 possible reasons (e.g., inadequate staffing), to why nursing measures was missed, the answers are given on a four-point Likert scale ranging from “significant cause” to “not a cause”. The MISSCARE Survey also covers data such as job satisfaction and intention to leave.

Background data regarding patients, including socio-demographic data, will be collected through a study-specific questionnaire. All questionnaire data will be processed in accordance with the instructions for each respective instrument. Non-parametric models will be used to identify differences between groups.

### Step 2

#### “Interacting” (Observation points 1 and 2)

**Fig 2:**
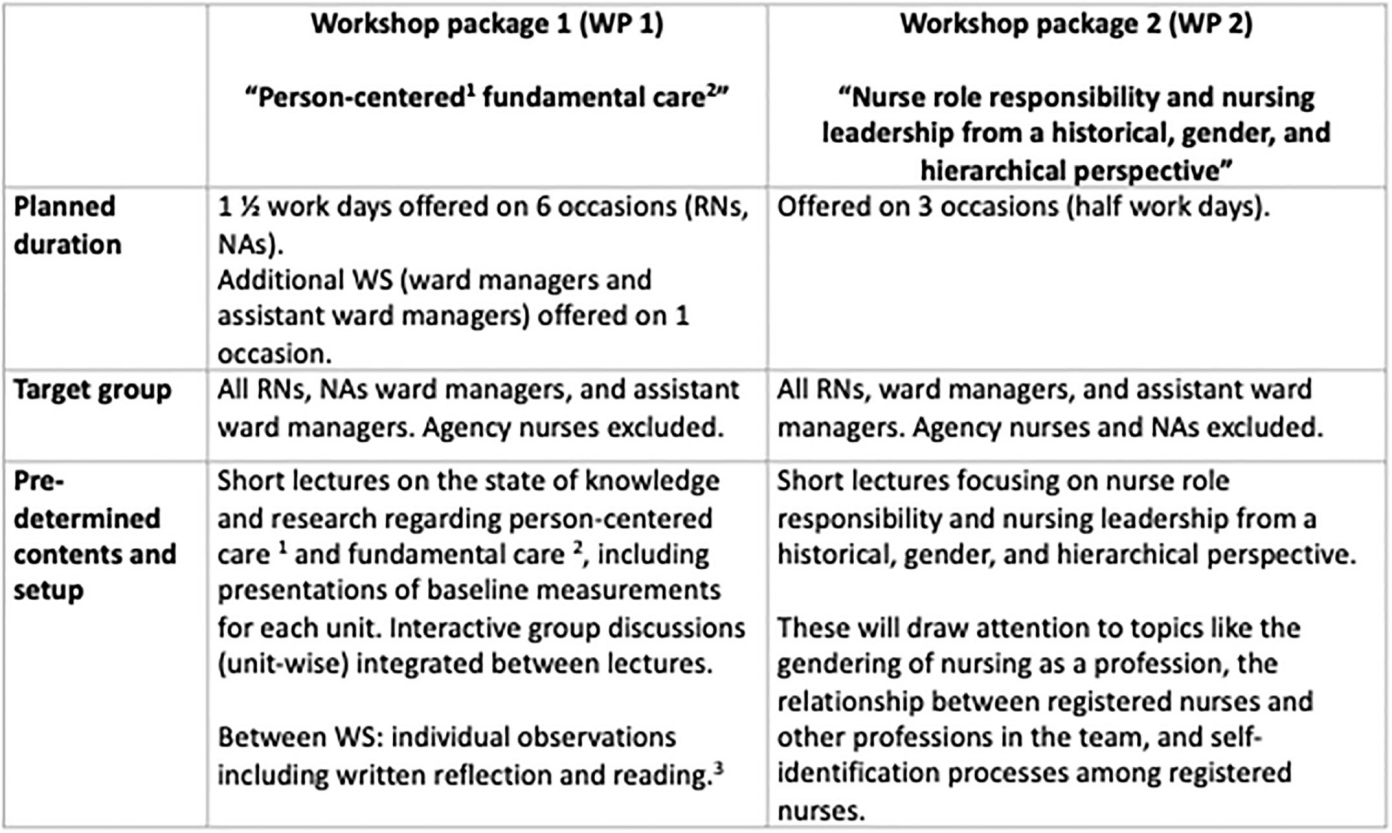
Description of workshop content during step two. 1. According to the Person-Centered Nursing Framework (McCormack & McCance, 2021). 2. According to the Fundamentals of Care framework (A. L. Kitson, 2018). 3. In the WS, the Swedish textbook on Fundamentals of Care will be used (Muntlin & Jangland, 2020).

Post-baseline and one year later, the care units will be presented with two workshop (WS) packages (WPs), followed by data collection. The structure and planned contents are shown in Fig 2.

#### Workshop package 1 (involves two workshops, WS I and WS II)

The purpose of the first workshop package (WP 1) (see Fig 2) is to open up and stimulate the surgical care units for a change towards person-centred fundamental care. The aim is to educate and start thought processes among the staff regarding person-centred fundamental care and person-centredness in connection to their own professional practices, including roles and responsibility. Another purpose is to gain further understanding of the contextual factors that enable or hinder the delivery of person-centred fundamental care and enable cocreation of relevant interventions at the care units.

WS I will be designed as one work day, directed at NAs, RNs, nurse managers, and assistant ward managers. The detailed content in WS 1will be designed by the research team with input from the group of facilitators but one of the pre-determined components in WS 1 is to present each care unit with the results of the baseline measurements described above, and reflect on their meaning. This approach will provide nursing stakeholders with important feedback from the patient perspective focusing on strengths and resources as well as possible areas of improvement. During WS 1 short lectures will be interspersed with interactive group discussions and written assignments related to the present state of person-centred fundamental care in the surgical care units. In addition, participants (NAs and RNs) will be instructed to perform a clinical observation focusing on fundamental care before WS 2. Fieldnotes will be taken by the facilitators during WS 1 and will be used to design the subsequent WS.

The second workshop (WS 2) (a half day) will be created in collaboration with the facilitators based on the reflections from WS 1 but one of the pre-determined components in WS 2 is to intercept the participants reflections regarding the clinical observations they performed since WS 1, and participants will also be asked to provide their written reflections. These reflections will be the starting point for discussions on the necessary future interventions.

WS 1 and 2 will be held at three times respectively, in order to involve the entire working group and avoid interfering with the daily clinical practice.

#### Workshop package 2

The second WP (WP 2) will be designed as a half work day (also held at three times respectively) directed at RNs, nurse managers, and assistant ward managers. The knowledge about contextual factors gained by WP 1 will be used in the design of the final content in collaboration with the facilitators. WP 2 will inform the later interaction process and contribute with an understanding on how the RNs and nursing leaders understand their day-to-day profession. The sense of meaning and control in relation to one´s work role is of high importance in the empowerment process of employees. Thus, the main purpose of WP 2 is to draw attention to nurse role responsibility and nursing leadership from a historical, gender, and hierarchical perspective through short lectures held by a historian within medical humanities. Group discussions will then be held related to the lectures. The lectures will cover topics such as the historical view of nursing and nurses, gendering, structures of power, the professionalization of nursing, and the direct impact on patient care.

#### Data collection and analysis step 2

During WP 1 data will be generated through the written assignments and reflections and the facilitators fieldnotes. In addition, following WP 1, focus group interviews will be held on three occasions to collect data. Interviews will be held at each care unit separately in order to identify possible interventions related to person-centered fundamental care. The results from WP 1 including suggested nursing interventions, will be presented back to the focus groups. The participants will be asked if there is anything they would like to change or add to the results and asked to review and discuss them in terms of relevance and feasibility. The groups will have a minimum of 3 participants and a maximum of 8 participants. The interviews and the written assignments/ reflections will be transcribed verbatim and undergo content analysis [37–39]. The analysis will be conducted by the research team and later discussed with the group of facilitators, the ward managers and the assistant head of department, both as a method of triangulation and as a mean to improve future implementation.

During WP 2, data will be collected through repeated group discussions. These discussions will be recorded and transcribed verbatim by the research team. Data will undergo qualitative content analysis followed by a gender analysis using Connell´s four dimensions of gender [40]. This analysis will provide knowledge of the challenges in modern nursing as well as identify discourses that work against the aims and goals of the future interventions and those that need to be strengthened, giving stakeholders power over the changes in discourse that need to be made actively during later interventions.

In addition, following the final WS, all participants will be asked to answer a short study-specific questionnaire regarding their knowledge of PCC, fundamental care and the role of the nurse. The data collection performed in the initial step (fundamental care needs, person-centered climate, and MNC) will also be repeated with the same number of participants for comparative analyses. Non-parametric models will be used to identify differences between groups. Repeated measurements will undergo analysis of variance (ANOVA).

### Step 3

#### Cocreating interventions to achieve person-centred care (Observation point 3 and beyond)

In the third step, the data collected during the previous steps will be used to reflect and determine the future course of the project, as well as the specific interventions to be conducted at each care unit to implement person-centred fundamental care. The focus will be to overview and summarize all stakeholders’ perspectives. The results will be used by the research team and nursing stakeholders to design and suggest interventions specific to each care unit. To increase the commitment, this will be a process where the care units are given the opportunity to direct and have control over the respective projects.

At this point, findings and suggested interventions from steps 1–2 will also be presented to the nursing management and the assistant head of the department. Shared resources available for future interventions will be identified and agreed upon, to ensure that the implementation process will be monitored and evaluated. The facilitators will be involved continuously in all relevant components of the studies, e.g., ethical considerations, participants, duration, intervention content, and relevant instruments.

#### Data collection and analysis step 3 (Observation point 3 and beyond)

The data collection performed during steps 1 and 2 (fundamental care needs, person-centred climate, and MNC) will be repeated. Non-parametric models will be used to identify differences between groups. Repeated measurements will undergo ANOVA and interviews will be analyzed descriptively and by summative content analysis [33]. Depending on the reflections made by the research team concerning the context, additional instruments or methods may be added at this point, in accordance with the AR method.

## Discussion

The aim of this project is to demonstrate the value of person-centred fundamental care and achieve a far- reaching change in the direction of nursing in surgical care units by using AR. The research will build upon past results and focus on cocreating innovations in practices and organizations. A reflective process and ownership in the nursing profession are expected to increase the value placed on nursing and strengthen nursing leadership at the units, preventing premature departure from the profession. Furthermore, this direction of nursing at the units, including improved utilization of RNs’ competence, is expected to stimulate person-centred fundamental care delivered to patients.

In a situation where RNs’ competence is not fully utilized, and RNs are prone to quit, due to restricted professional autonomy [10], researchers need to collaborate with the nursing profession to design new far-reaching interventions. The AR design is considered to be appropriate for such interventions, as it helps researchers gain a deeper understanding of the contextual factors important for ongoing or forthcoming interventions. Furthermore, letting the data from the different steps build on each other, e.g., the results from patients being presented to nursing stakeholders, is expected to support and stimulate motivation for change.

In this case and the present setting, the AR design has several advantages. However, this type of design creates challenges due to its multimodal and time-consuming nature. It has been suggested that the use of standardized measurement tools and a predetermined framework can be helpful in overcoming some of these challenges [26]. Thus, the present research protocol and an estimation of the timeline are vital. The collaboration with the nursing leadership and nursing representatives, established in line with the AR design, help the research team be prepared for issues that may occur in clinical practice and require adaptation during the process. The collaborative meetings will be built on openness and collective decisions, but to ensure objectivity, the research team will also hold its own meetings every other week, to discuss both practical and ethical issues throughout the research process.

The research program has its ontological foundation in nursing. However, the research integrates the perspectives of nursing science and medical humanities. Collaboration across these disciplines is a unique way of studying the phenomenon. These different perspectives will provide knowledge and uncover the various and conflicting pressures that work at individual, organizational, and institutional levels to affect the recruitment, retention, and turnover of nurses in a hospital setting, including the role and responsibility of the nursing leadership. The combination of WS being planned for, participants’ reflective notes on their daily practices, and focus group interviews serves to open for critical thinking regarding how the nurse profession has been and is being shaped by historical factors, as well as gender factors impacting on nurses and nursing leadership priorities today [41]. The value and requirements of person-centered fundamental care, and the organizational components that hinder or facilitate such care, together with participants’ discussions regarding the historical development of the nursing role, will thus inform researchers and stakeholders on which interventions are needed. The i-PARISH framework will be used preparing for innovations in clinical practice to be implemented. The framework highlights facilitation and a supporting and engaged leadership as pivotal in implementation of new evidence in clinical practice [42].

### Strengths and limitations

One strength of this research project is that it is based on cocreation of knowledge derived from practical problems in clinical practice on the surgical care units. The research questions grew from a collaborative effort between the researchers and clinicians, focusing on urgent challenges in daily practice. Several possible limitations have been identified. One limitation is the single setting. Although the research program is performed at one surgical department and one university hospital, it is our strong conviction, based on clinical and research experience, that the knowledge gleaned will be relevant to other hospital settings and have good transferability.

The high turnover of nurses at the department could prevent the identified interventions from being implemented and decrease the sustainability of changes. Thus, turnover may impact on the procedure and outcome. However, the data collection will not be dependent on any specific nurse or nursing leader. Based on these potential limitations, the research team will need to have a critical gaze in considering the future interventions or changes to be implemented and what data are to be collected [26]. Feasibility needs to be high on the agenda when considering interventions in collaboration with the nursing management and stakeholders in clinical practice. Though large-scale projects may seem attractive, the implementation of smaller changes can have greater potential to succeed and provide RNs and nursing leaders with unit-specific results to continuously reflect on, act upon, and refine.

## Supporting information

S1 Checklist SPIRIT 2013 checklist: Recommended items to address in a clinical trial protocol and related documents*. (DOC)
