# Supplementary material for: InCHARGE: Co-creating, implementing and evaluating interventions to utilize nurses’ competence and achieve person-centred fundamental care—A research protocol describing an action research approach
Source: PLoS One. 2024 Jul 2;19(7):e0304700. doi: 10.1371/journal.pone.0304700 (PMC11218940; doi:10.1371/journal.pone.0304700)
Supplement: S1 Checklist — (DOC) [file pone.0304700.s001.doc]

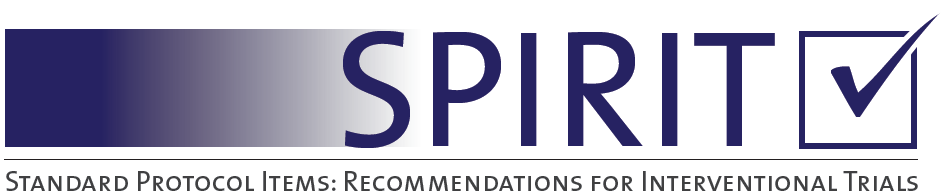


SPIRIT 2013 Checklist: Recommended items to address in a clinical trial protocol and related documents*

| Section/item | ItemNo | Description |
| --- | --- | --- |
| **Administrative information** | | |
| Title | 1 | “inCHARGE: Cocreating, implementing, and evaluating interventions to utilize nurses’ competence and achieve person-centered fundamental care by an action research approach – a research protocol.” |
| Trial registration | 2a | The trial is registered in a public study register (Public360 DNR 2023-00042) Date of registration 230313. |
|  | 2b | All items from the World Health Organization Trial Registration Data Set   1. Public360 DNR 2023-00042 2. 230313 3. The Swedish Ethical Review Authority (DNR 2022-01557-01) 4. No external funding. Please see attached funding letter 5. No external funding. Please see attached funding letter 6. No external funding. Please see attached funding letter 7. [anna.hauffman@surgsci.uu.se](mailto:anna.hauffman@surgsci.uu.se), +46703381353,   Department of Surgical Sciences, Uppsala university 751 85 Uppsala, Sweden   1. Anna Hauffman, PhD, senior lecturer (contact information above)   Eva Jangland, PhD, associate professor, senior lecturer, +4618-6113722  Department of Surgical Sciences, Uppsala university 751 85 Uppsala, Sweden   1. The in Charge program 2. “inCHARGE: Cocreating, implementing, and evaluating interventions to utilize nurses’ competence and achieve person-centered fundamental care by an action research approach – a research protocol.” 3. Sweden 4. No specific health conditions will be studied 5. In this long-term study, the action research design will be used thru three main phases: “Getting to know the context,” “Interacting,” and “Cocreating interventions.” During the process the research team will work with empowerment in relation to work role as the primary method in collaborations with stakeholders.The duration of the phases will be approximately five years from ethical approval of the first phase. The results from each phase will be used with reflexivity to shape the subsequent phase. The project will be theoretically grounded in person-centered fundamental care The i-PARISH framework will be used as a tool to guide the collaboration with stakeholders in all phases. 6. The stakeholders to be invited to participate include patients being cared for at three surgical care units, RNs (including specialist nurses and nurse practitioners, excluding agency nurses), NAs, ward managers, and assistant ward managers employed at the care units. Regarding patient stakeholders, all eligible patients aged 18 years or older being cared for at the units on the predetermined days of measurements will be consecutively invited to participate by members of the research team. The exclusion criteria are cognitive impairment, infectious disease, Karnovsky performance status < 20, or inability to speak and understand Swedish. As for nursing stakeholders, all RNs (n=60), NAs (n=64), ward managers and assistant ward managers (n=10) employed in the included departments will be invited to participate in conjunction with staff meetings. 7. Action research design including repeated measures. 8. The enrolment started in 2022 after approval from the Swedish Ethical Review Authority. |
| Protocol version | 3 | 1. Not applicable 2. Recruiting 3. The primary outcomes are person-centered climate measured by the validated Swedish version of the person-centered climate questionnaire and missed nursing care measured by the Swedish version of the MISSCARE Survey. The measures will be repeated at four occasions (the exact timepoints will depend on the phases in the study). Data collection is planned to be terminated in 2025. 4. Data regarding patients’ fundamental care needs will be collected by structured interviews at the start and the end of the study (patientstakeholders). Additional qualitative data will be collected by focus group interviews at two occasions (nursing stakeholders). 5. The Swedish Ethical Review Authority approval was announced 2022-04-12 (DNR 2022-01557-01) 6. Data collection is planned to be terminated in 2025. 7. Not applicable 8. Not planned at the moment.   See date of submission, (the first version of the protocol). |
| Funding | 4 | No external funding. Please see attached funding letter |
| Roles and responsibilities | 5a | All authors have contributed to the protocol, please see submission*.* |
|  | 5b | Not applicable |
|  | 5c | Not applicable |
|  | 5d | Not applicable |
|  |  |  |
| **Introduction**  Background, rationale and objectives | 6a-b, 7 | The overarching aim of this long-term project is to achieve a change in the direction of nursing and nursing leadership, in collaboration with nursing stakeholders, and to identify and cocreate interventions to stimulate person-centered fundamental care in surgical settings. A further aim is to evaluate the effects of the project regarding PCC, MNC, and the fulfillment of patients’ fundamental care needs in surgical care units. The final interventions will be decided and owned by the nursing profession itself with the support from the research team. It is expected to increase the value placed on nursing and strengthen nursing leadership and stimulate person-centred fundamental care delivered to patients at the units included in the research project. |
| Trial design | 8 | Participatory action research with repeated measures. |
| Methods: Participants, interventions, and outcomes | | |
| Study setting | 9 | The project will be undertaken at the surgical department of a Swedish university hospital comprising three care units, where patients are cared for following acute or planned admission due to vascular, endocrine, colorectal, esophageal/ventricle, or liver/bile/pancreatic illness, trauma, or transplantation.  The stakeholders to be invited to participate include patients being cared for at the units, RNs (including specialist nurses and nurse practitioners, excluding agency nurses), NAs, ward managers, and assistant ward managers employed at the care units (n=134). |
| Eligibility criteria | 10 | All eligible patients aged 18 years or older being cared for at the units on the predetermined days of measurements will be consecutively invited to participate by members of the research team. The exclusion criteria are cognitive impairment, infectious disease, Karnovsky performance status < 20, or inability to speak and understand Swedish. The interventions will be carried out in a collaboration between the research team and the stakeholders. All eligible staff, excluding agency nurses will be invited to participate. The goal is to include 100 patients and 100 nursing staff. |
| Interventions | 11a | During the action research process (approximately five years long) the research team will work with empowerment in relation to work role as the primary method in collaborations with stakeholders. The research process is divided into three phases “getting to know the context”, "interacting” and “cocreating interventions”. The first phase contains no intervention while in the second, the care units will be presented with two workshop packages followed by data collection. The workshops will start with interactive evidence-based education on person-centered approach and fundamental care guided by the FoC framework. This is intended to start thought processes among the staff regarding person-centered fundamental care in connection to their own professional practices, including roles and responsibility. For the second workshop the research teams knowledge on contextual factors from the first workshop will be used in the design of the final content. The main purpose of this workshop is empowerment by attention to nurse role responsibility and nursing leadership from a historical, gender, and hierarchical perspective. The workshop will inform the later interaction process and contribute with an understanding on how the RNs and nursing leaders understand their day-to-day profession. The final interventions to improve person-centered care in the care units will be decided and developed in collaboration with the stakeholders in the third phase. |
|  | 11b | Not applicable |
|  | 11c | Not applicable |
|  | 11d | Not applicable |
| Outcomes | 12 | The main outcomes are person-centered climate and missed nursing care. The person-centered climate will be measured with the validated Swedish version of the person-centered climate questionnaire (staff version and patient version). The Swedish version of the MISSCARE Survey will be used to measure how often and why staff are not able to perform various nursing care measures (staff only). These measures will be carried out at baseline and at two more occasions during 2023-2025. It will be collected from the staff currently working in the care units at the time and from patients being cared for at the time.  In addition, qualitative data will be collected thru observations and interviews following e.g workshops. During the collection of questionnaires in the patient group, approximately 30 patients will be asked if they want to answer structured interview questions regarding the fulfillment of fundamental care needs during their time at the care units. These data will be collected by fieldnotes only. |
| Participant timeline | 13 | Data will be collected repeatedly; the exact time frame will be dependent on the progress of the project as a whole. For example, the second observation point will take place after the workshops. The third observation point will take place before the launch of the staff-driven interventions. The last observation point is estimated to be in 2025. |
| Sample size | 14 | At the three care units there are 134 possible nursing staff to be included and the goal is to obtain questionnaires for at least 70 % (n=100) of those. The goal for patient participants is the same (n=100). |
| Recruitment | 15 | All eligible staff and patients will be approached directly by members of the research team. Data will be collected directly after the informed consent is signed. |
| **Methods: Assignment of interventions (for controlled trials)** | | |
| Allocation: |  |  |
| Sequence generation | 16a | Not applicable |
| Allocation concealment mechanism | 16b | Not applicable |
| Implementation | 16c | Not applicable |
| Blinding (masking) | 17a | Not applicable |
|  | 17b | Not applicable |
| **Methods: Data collection, management, and analysis** | | |
| Data collection methods | 18a | Person-centered climate measured by the validated Swedish version of the person-centered climate questionnaire, staff version (Edvardsson, Sandman, & Rasmussen, 2009) and patient version (Edvardsson, Sandman, & Rasmussen, 2008). Both questionnaires consist of 14 items with answers given on a six-point Likert-scale, from 1 = “Disagree completely” to 6 = “Agree completely.” The questionnaires will measure person-centered climate based on three factors: climate of safety, climate of everydayness, and climate of community.  The Swedish version of the MISSCARE Survey (Kalisch & Williams, 2009) will be used to measure how often and why staff are not able to perform various nursing care measures. The first part consists of 24 nursing measures (e.g. oral care) with answers given on a five-point Likert-scale, from “always carried out” to “never carried out”. The second part consists of 17 possible reasons (e.g. inadequate staffing), to why nursing measures was missed, the answers are given on a four point Likert scale ranging from “significant cause” to “not a cause”.  The MISSCARE Survey also covers background data such as job satisfaction and intention to leave. Background data regarding patients, including socio-demographic data, will be collected through a study-specific questionnaire. |
|  | 18b | Not applicable |
| Data management | 19 | Data will be collected thru questionnaires and interviews, no personal information such as social security number or name will be gathered in any of the material. All questionnaire data will be locked in a safe cabinet. Recordings will be taken on a Dictaphone (no internet connection) and kept locked in a safe cabinet. All interviews will be transcribed and the audio files erased from the Dictaphone. The transcripts will not reveal the identity of the informants. |
| Statistical methods | 20a | All questionnaire data will be processed in accordance with the instructions for each respective instrument. Non-parametric models will be used to identify differences between groups |
|  | 20b | Not applicable |
|  | 20c | If a participant has more than 50 % missing values in a questionnaire listwise deletion will be performed. |
| **Methods: Monitoring** | | |
| Data monitoring | 21a | The type of data collected during this action research process with empowerment as the primary mechanism of action does not call for external monitoring. |
|  | 21b | Not applicable |
| Harms | 22 | No adverse events are expected, any unintended effects will be presented in the following publications. |
| Auditing | 23 | Not applicable |
| Ethics and dissemination | | |
| Research ethics approval | 24 | No further approval than the swedish ethical review authority approval (DNR 2022-01557-01) is planned for. |
| Protocol amendments | 25 | Any deviation from the protocol will be announced in forthcoming publications (referring to the protocol). |
| Consent or assent | 26a | The members of the research team will handle the informed consents that will be kept locked in a safe cabinet. |
|  | 26b | Not applicable |
| Confidentiality | 27 | No personal data such as social security number will be collected, the patient stakeholders in the study will be identified at the care units and contacted first by staff (to ask them if they would like to talk to a member of the research team) and not until then approached by the research team. Information about the participants name and the informed consent forms will be kept apart from all questionnaires and locked in a safe cabinet. The questionnaires will be assigned a code number and the code key kept apart from questionnaires, locked in a safe cabinet. The research team that is not involved in gathering data will only work with data involving the code number. The collected data will be preserved for at least 10 years in accordance with Swedish legislation. |
| Declaration of interests | 28 | Not applicable |
| Access to data | 29 | Only the research team will have access to raw data but datasets may be shared with other researchers upon request. |
| Ancillary and post-trial care | 30 | Not applicable. |
| Dissemination policy | 31a | The results will be communicated by publications (following per review) and by reporting back to the nursing stakeholders at involved care units. |
|  | 31b | The research team use the Vancouver recommendations for authorship. No professional writers will be used. |
|  | 31c | Not applicable |
|  |  |  |
| Informed consent materials | 32 | Consent forms and other related documentation have all been approved by the Swedish ethical review authority |
| Biological specimens | 33 | Not applicable |

*It is strongly recommended that this checklist be read in conjunction with the SPIRIT 2013 Explanation & Elaboration for important clarification on the items. Amendments to the protocol should be tracked and dated. The SPIRIT checklist is copyrighted by the SPIRIT Group under the Creative Commons “[Attribution-NonCommercial-NoDerivs 3.0 Unported](http://www.creativecommons.org/licenses/by-nc-nd/3.0/)” license.
